# MUMmer4: A fast and versatile genome alignment system

**DOI:** 10.1371/journal.pcbi.1005944

**Published:** 2018-01-26

**Authors:** Guillaume Marçais, Arthur L. Delcher, Adam M. Phillippy, Rachel Coston, Steven L. Salzberg, Aleksey Zimin

**Affiliations:** 1 Institute for Physical Science and Technology, University of Maryland, College Park, Maryland, United States of America; 2 Computational Biology Department, Carnegie Mellon University, Pittsburgh, Pennsylvania, United States of America; 3 Center for Computational Biology, Johns Hopkins School of Medicine, Baltimore, Maryland, United States of America; 4 National Human Genome Research Institute, Bethesda, Maryland, United States of America; 5 Departments of Biomedical Engineering, Computer Science, and Biostatistics, Johns Hopkins University, Baltimore, Maryland, United States of America; University of Technology Sydney, AUSTRALIA

## Abstract

The MUMmer system and the genome sequence aligner nucmer included within it are among the most widely used alignment packages in genomics. Since the last major release of MUMmer version 3 in 2004, it has been applied to many types of problems including aligning whole genome sequences, aligning reads to a reference genome, and comparing different assemblies of the same genome. Despite its broad utility, MUMmer3 has limitations that can make it difficult to use for large genomes and for the very large sequence data sets that are common today. In this paper we describe MUMmer4, a substantially improved version of MUMmer that addresses genome size constraints by changing the 32-bit suffix tree data structure at the core of MUMmer to a 48-bit suffix array, and that offers improved speed through parallel processing of input query sequences. With a theoretical limit on the input size of 141Tbp, MUMmer4 can now work with input sequences of any biologically realistic length. We show that as a result of these enhancements, the nucmer program in MUMmer4 is easily able to handle alignments of large genomes; we illustrate this with an alignment of the human and chimpanzee genomes, which allows us to compute that the two species are 98% identical across 96% of their length. With the enhancements described here, MUMmer4 can also be used to efficiently align reads to reference genomes, although it is less sensitive and accurate than the dedicated read aligners. The nucmer aligner in MUMmer4 can now be called from scripting languages such as Perl, Python and Ruby. These improvements make MUMer4 one the most versatile genome alignment packages available.

This is a *PLOS Computational Biology* Software paper.

## Introduction

Since the 2004 publication of the MUMmer3 sequence alignment package [1], the bioinformatics landscape has changed dramatically. The cost of generating sequence data has decreased rapidly, leading to an exponential increase in the number of assembled genomes and a proliferation of sequencing-based assays. Along with these increases came a corresponding increase in the demand for efficient sequence alignment algorithms. Applications of alignment include resequencing humans to discover single nucleotide polymorphisms (SNPs), sequencing and comparison of different species to detect evolutionarily conserved elements, alignment to detect large-scale chromosomal rearrangements, and more. Alignment algorithms are also used to create and validate genome assemblies and to compare them from one version of a genome to the next. These and other applications motivate the need for fast and reliable sequence alignment techniques that are capable of handling large genomes and large volumes of sequence data. Although raw computing speed has not kept pace with improvements in sequencing efficiency, improvements in memory capacity and parallel processing can be used to compensate; in particular, algorithms can demand larger amounts of random-access memory (RAM) and multiple cores to handle the challenges of larger genomes and data sets.

Many DNA and protein sequence alignment software packages are available today, including BLAST [2], Bowtie [3], BWA [4], Blat [5], Mauve [6], LASTZ [7], and BLASR [8]. Some of these systems target a particular type of alignment problem, *e.g.*, BWA and Bowtie2 are best suited for aligning large numbers of relatively short sequences (50-300 bp) to a reference genome; and BLASR is designed to align long high-error-rate (15-20%) sequences to a reference. MUMmer and its sequence alignment component nucmer were originally developed [9] for alignment of whole bacterial genomes to other genomes, but they have evolved into general purpose aligners that remain very widely used. In addition to aligning whole genomes, MUMmer3 is capable of aligning short and long reads with variable error rates to a reference genome, but it is inefficient in doing so. It is not restricted to DNA and can also align protein sequences. MUMmer produces only pairwise genome alignments; i.e., it is designed to compute alignments of pairs of DNA sequences as opposed to multi-alignments of many genome sequences. The very large data sets produced by current sequencing technology, though, sometimes exceed MUMmer3’s limitations on the maximum length of its input sequences, and the scope of these data also require ever-longer run times. To address the runtime challenge, one can use wrapper scripts to break long sequences into smaller ones and run multiple, parallel MUMmer3 jobs in batches. However, such *ad hoc* parallelization is both inefficient and inconvenient, requiring additional steps to combine and process the resulting multiple outputs.

In this paper, we describe MUMmer4, a major new release that has been re-engineered and extended based on MUMmer3, providing full backward compatibility of inputs, outputs and options. The biggest changes affect the nucmer sequence aligner that is part of MUMmer4, and most of our discussion will focus on these. Although the executables in both MUMmer3 and MUMmer4 are called “nucmer”, for clarity we will use nucmer3 to refer to the nucmer program in MUMmer3, and nucmer4 to refer to the MUMmer4 version. Unlike nucmer3, which has limits of ∼500 Mb on the reference sequence size and ∼4 Gb on the query sequence size, nucmer4 can process sequences of essentially any size, with a theoretical limit of 141 trillion bases (Tbp), due to its use of a new 48-bit long suffix array instead of a 32-bit suffix tree. It is unlikely that this limit, which is 1000 times larger than the largest known genome, will be exceeded in any current practical scenario. Furthermore, nucmer4 can use multiple cores on the same computer, resulting in much faster run times. In addition, while nucmer3 computes the suffix tree on the fly–at the time the program is invoked–and discards it after each use, nucmer4 offers a more efficient two-step mode of operation, similar to short read aligners, where the suffix array of the reference can be created and saved to disk, and then loaded repeatedly to align sets of query sequences. Nucmer4 produces output that is identical to nucmer3, except for the order in which the alignments are output. In the results below, we demonstrate nucmer4’s improvements in speed by comparing it to nucmer3 and to other aligners, showing that this multi-purpose sequence aligner compares favorably even with aligners that have been optimized to handle more specialized alignment tasks.

The input and output formats of nucmer4 have also been changed to make it compatible (optionally) with software pipelines that are designed to operate on next-generation sequencing (NGS) reads. On the input side, nucmer4 now accepts both FASTA and FASTQ sequence formats. On the output side, nucmer3 only provided output in a custom format known as a delta file, which can be used by other MUMmer utilities to produce alignments, SNP reports, and plots. Nucmer4 can now produce either a delta file or a SAM file, the most common output format for short read aligners. SAM output is compatible with many other packages, including SAMtools [10].

## Design and implementation

### MUMmer3 implementation

The main pipeline of MUMmer is NUCmer, to align nucleotide sequences. The NUCmer pipeline consists of 4 main programs driven by the nucmer Perl program: (1) preprocessing of the input sequences (the prenuc program), (2) finding exact seed matches (the mummer program), (3) clustering of matches (the mgaps program), and (4) extending and joining matches (the postnuc program).

The prenuc program transforms the multi-fasta file of the reference sequence into a valid format for the mummer program: a single string using x characters as a separators.

The mummer program finds exact matches between pair of sequences that are maximal in length and at *l* bases long. By default, mummer finds all matches between a query sequence and a reference sequence that are unique in the reference, called Maximal Unique Matches (MUMs). A command-line option allows mummer to instead find all matches that are unique in both the query and the reference, or to find all Maximal Exact Matches (MEMs) regardless of uniqueness. The “MEM” option should be used with care, as it can produce very large amounts of output when the input sequences contain numerous repeats. In MUMmer3 and earlier versions, this exact match step is accomplished using a suffix tree data structure [9].

The MUMs or MEMs are then piped into the mgaps program which groups the exact matches into clusters based on their proximity to one another. In a typical alignment run, many of these exact matches will become part of longer inexact matches, which will be computed in the next step. mgaps writes its output to a file.

Finally, the postnuc program computes longer alignments from the clustered exact matches. It first re-reads the reference sequence to record the names as well as the beginning and ending positions of the original reference subsequences in *S*.

Then the postnuc program uses a banded Smith-Waterman alignment algorithm [11] to find alignments between consecutive exact matches within each cluster and to extend the ends of the first and last matches of each cluster. It writes the resulting alignments to disk, after translating the coordinates from positions in the string *S* back to positions in the reference subsequences.

If this implementation has the advantage of being modular, it suffers from a number of inefficiencies and limitations: (1) the reference sequence is read twice, which creates more I/O, duplicated work, and prevents streaming in the reference; (2) multiple intermediary steps write their results to disk, creating extra I/O; (3) the pipeline is hard to use from another program, written in C or script language.

### MUMmer4 implementation

We have designed the nucmer4 program to overcome many of the limitations of the nucmer3 pipeline. It avoids the redundant work done by both prenuc and postnuc, parsing the reference sequence only once, thus allowing it to take input from a pipe. It also avoids the use of the file system or pipes to pass information between stages by instead passing C++ objects representing the matches and clusters. This re-design not only provides speed advantages, but it makes it far easier to incorporate nucmer4 into other software packages.

In nucmer4, we implemented the primary computational steps of each component program into a C++ library, named libumdmummer. Each step in the original nucmer3 pipeline is now represented by a C++ class. Although the individual programs from the pipeline are still available in the MUMmer4 software distribution, the nucmer program is now a unified, multi-threaded C++ binary that uses the MUMmer library.

The biggest internal change to MUMmer4 is the use of a suffix array instead of a suffix tree to index the reference sequences. Although both suffix trees and suffix arrays require a linear amount of memory to represent the reference sequences, the constant factors are smaller with (carefully implemented) suffix arrays, reducing the total memory requirement. To make the switch, we utilized the essaMEM program [12], which was designed as a drop-in replacement for MUMmer. The MUMmer4 code includes a wrapper around the essaMEM code that replaces the original suffix tree-building code.

In addition, we replaced the qsufsort [13] suffix array construction code in essaMEM by the faster library divsufsort [14]. After building the suffix array, we then parse the query sequences (nucmer allows the query to be a multi-fasta or multi-fastq file containing any number of sequences) and pass them one by one to worker threads using a lock-free first-in first-out (FIFO) queue. Each worker thread computes the exact alignments, clusters them, and runs the banded Smith-Waterman alignment routine (performed by postnuc in nucmer3) for its single query sequence.

The results are output to disk or to stdout, again using a lock-free FIFO queue to synchronize writing by the different threads. The default output appears in delta format and is designed to be identical to the output of Nucmer3. Because of the multi-threaded operation, the order of the sequences in the output file is not strictly preserved, and may be different from run to run. Thus, although the alignments of nucmer4 and nucmer3 are identical, the order in which they appear in the output files might differ.

### Query parallelism

One of the most common alignment tasks today is to align many sequences against a reference genome; *e.g.*, aligning a large set of reads to the human genome. In MUMmer4, we introduce parallelism at the level of these query sequences, allowing many of them to be aligned simultaneously against a reference. When aligning large numbers of query sequences, nucmer can achieve a high level of concurrency, limited only by the number of processing cores. On the other hand, if the query contains a small number of large sequences (*e.g.*, entire chromosomes), the concurrency is limited by the number of sequences in the query. In the limiting case of aligning a single query to the reference, query parallelism provides no benefit.

### Changes to built-in size limits

One of the built-in limitations in MUMmer3 is a strict upper bound on the lengths of the input sequences. The implementation limits the total reference sequence—*i.e.*, the length of the concatenated string *S*—to ∼536 Mb. This is large enough for all bacterial genomes and for some small eukaryotes, such as *Drosophila melanogaster*; however, it is not sufficient for larger plant and animal genomes, which often exceed 1 Gb and can be as large as 32 Gb as seen, for example, in the sugar pine, *Pinus lambertiana* [15]. MUMmer3 also limits the total size of the query sequences to 2^32^ − 1 ≈ 4 Gb.

MUMmer4 significantly raises these size limits, making it possible to align even the largest genomes. The length of the reference sequence for each alignment job is limited in principle to 2^47^ − 1 ≈ 141 Tb. The available computer hardware provides a more practical limit; MUMmer4 uses 15 bytes per base of the reference sequence to store the index. Thus for example, if the reference sequence were 66 Gb (equal to 22 human genomes), the suffix array would require about 1Tb of computer RAM. To accommodate computers with limited RAM, MUMmer4 includes a new “--batch X” option that automatically splits long reference sequences and processes them in smaller batches. Specifically, it loads X bases of reference sequence, matches all query sequences against the first batch, then loads the next X bases of reference and so on. In practice this eliminates any restrictions on the size of the input reference sequence and also allows the user to tune the alignment runs to the total available computer memory. MUMmer4 has no absolute limit on the total query sequence size. Also, as a minor improvement, MUMmer4 removes the previous 128-character limit on the length of the names of reference and query sequences.

### Saving a large index to disk

MUMmer4 now includes options to save and load the suffix array for a given reference. The most popular systems for aligning reads to a reference genome, Bowtie2 [16] and BWA [4], both assume that their index (a Burrows-Wheeler transform using the FM index) has been pre-computed and stored in a file, which allows the alignment step to run much faster. Suffix array construction is primarily a single-threaded task that can take about 36 minutes for a 3 Gb genome. Many large genomes—*e.g.*, the human genome, the mouse genome, and other model organisms—are essentially static, with periodic updates and controlled versions. Thus there is no need to build the suffix array on the fly (at the time of alignment) if one intends to use the same reference repeatedly, *e.g.*, to align Illumina data to a human reference genome. Using the new MUMmer4 option, the suffix array can be built once, saved and then loaded for each run.

For a large reference genome, MUMmer4 uses approximately 13-14 bytes/base for the index (6 bytes per base for the suffix array, another 6 for the inverse suffix array, and 1 byte for the LCP array). For example, the size of the suffix array for the human genome is approximately 39 GB. Loading the suffix array from memory is much faster than constructing it from scratch, and on a typical server with 200 MB/sec input speed, a 39 GB suffix array will load in approximately 3 minutes.

Nucmer4 requires additional memory in scenarios when running with multiple threads on query sequences that are large. Our parallelization routine distributes multiple query sequences into multiple threads, one sequence per thread, and query sequences have to be loaded into memory. The step of loading multiple query sequences into memory at the same time increases peak memory usage in such scenarios, proportional to the number of threads used. With only one thread, memory usage is similar between nucmer3 and nucmer4.

The original output format of nucmer, the delta format, contains only the minimum information necessary to quickly recreate the alignment. It contains the name of the matching sequences, the length of the match, number of errors and positions of indels. An important addition in MUMmer4 is option to produce output in SAM format, one of the most widely used formats for alignments of NGS data [10]. This also allows the MUMmer4 output to be used in any of the numerous tools that require SAM files as input. Nucmer4 supports two different options for SAM format output. With --sam-short, nucmer4 reports only the name of the matching sequence, length, and CIGAR string (which reports the indel positions). With --sam-long, it additionally reports the MD string (which specifies the mismatching positions), the sequence and, if applicable, the quality values of the matching sequence. The long format is more expensive to compute and it generates larger output files, but this option allows nucmer4 to match the behavior of other aligners such as Bowtie2 or BWA.

We transformed the global variables in the original code to object instance variables. As a result, it is possible for an application using libumdmummer to instantiate multiple aligner objects concurrently, for example in a multi-threaded program. The new library is also usable from the scripting languages Python, Perl or Ruby, making MUMmer4 much more flexible. We used the SWIG [17] tool to generate the script bindings, allowing developers to create bindings to the many other languages supported by SWIG with little extra work. This binding allows a user to align a pair of sequences directly from the scripting languages, returning an array of the alignments. Examples of how to use the library in a C++ program or in a script are provided in the supplementary material (S1 File).

## Results

The enhancements in MUMmer4 allow it to align (1) a pair of genomes to each other, (2) two large sets of sequences to one another, or (3) a set of reads to a reference genome. This functionality stands in contrast to specialized NGS alignment tools such as Bowtie2, BWA or BLASR, which are designed for aligning reads to a reference genome. BWA and Bowtie2 are designed primarily for alignment of short Illumina sequences, while BLASR is designed for alignment of long high-error-rate sequences, such as those produced by the Pacific Biosciences SMRT and Oxford Nanopore MinION technologies. MUMmer4’s suffix array data structure ensures that alignment time is a linear function of the read length and is independent of the size of the reference genome, similar to NGS aligners that use the Burrows-Wheeler transform as their principal data structure.

Although specialized read aligners are more accurate and sensitive (and thus likely preferable) for that task, we show below that when run with default settings, the speed of MUMmer4 is comparable to Bowtie2, BWA and BLASR for the alignment of both short low-error-rate (Illumina) and long high-error-read (PacBio) sequences to a reference genome. MUMmer4 is much faster than Mauve [6] and LASTZ [7] aligners, built using similar data structures. Table 1 shows detailed feature comparison between MUMmer4 and the other aligners. MUMmer4 is a significant upgrade over MUMmer3 in terms of features. MUMmer4 is the only aligner that can be called from Perl, Python or C++. Unlike Mauve, LASTZ or Blat, it is multi-threaded. It is only available for Linux and users looking for Windows-compatible aligner with a nice GUI (Graphical User Interface) may be better off using Mauve. MUMmer4 does not compute P-values or E-values for its alignments, and thus Blast, LASTZ or BLASR are preferred over MUMmer4 if they are needed.

**Table 1:** Comparison of aligner features. A checkmark means the feature is present and usable, otherwise the feature is absent or its use is impractical. Features that are absent by design are marked with a dash.

Aligner	Graphical User Interface	Multi-platform Windows/Linux	Multi-threaded	Callable from C++, scripting languages	Whole genome aln.	Short read aln.	Long read aln.	SAM format output	P-value output
MUMmer4			✔	✔	✔	✔	✔	✔	
MUMmer3					✔				
Blast	✔	✔	✔		✔				✔
Blat					✔				✔
Mauve	✔	✔			✔				
LASTZ					✔			✔	✔
bwa-mem			✔		-	✔	✔	✔	
Bowtie2			✔		-	✔	-	✔	
BLASR			✔		-	-	✔	✔	✔

We also show substantial improvements in speed and versatility compared to the MUMmer3 package. In the Supplementary material, we report all settings and command line parameters used for generating the results shown here. All timings were measured on a dual-CPU, 32-core AMD Opteron 6276 computer with 256 GB of DDR3 PC3-12800 RAM.

### Data used

For the comparisons shown here, we used two different organisms as reference genomes. For mapping reads to a genome, we used the *Arabidopsis thaliana* Col-0 reference genome [18] and the human reference genome, version GRCh38.p7 [19]. We removed all alt sequences from the human reference sequence, because they could skew our human—chimpanzee genome to genome comparison statistics.

As a test of MUMmer’s performance on aligning large genomes, we aligned the reference assemblies of the human genome and the chimpanzee (*Pan troglodytes*) genome [20] (release PanTro4, GenBank accession GCF_000001515.6) to one another. To compare MUMmer4’s performance to MUMmer3 on aligning whole genomes, we provide the timing results for aligning the *Arabidopsis lyrata* assembly 1.0 [21] to the *A. thaliana* reference. Below we describe MUMmer4’s performance statistics on these tasks as well as summaries of the resulting alignments.

To benchmark MUMmer4’s performance on aligning reads to a reference genome, we performed two sets of experiments. First, we aligned PacBio SMRT and Illumina reads from *A. thaliana* Ler-0 [22], data available from [23], to the *A. thaliana* Col-0 reference [18]. Second, we aligned a subset of about 20x coverage Illumina and a subset of 10x coverage PacBio reads from the publicly available Ashkenazi data set (available from the Genome in a Bottle project [24], NCBI SRA accession SRX847862) to the human genome reference GRCh38.p7. Detailed information about these data sets is shown in Table 2.

**Table 2:** Description of the data sets used for aligner comparisons. The Illumina and PacBio data for *A. thaliana* is available from [23]; the human Illumina and PacBio reads are from the Ashkenazi child data set (available from the Genome in a Bottle project [24], NCBI SRA accession SRX847862). The reference genomes are the *Arabidopsis thaliana* Col-0 reference genome [18], the human reference genome version GRCh38.p7 [19], and the chimpanzee (*Pan troglodytes*) genome [20] (release PanTro4, GenBank accession GCF00001515.6).

Reference	Genome size	Illumina			PacBio		
		number of reads	bases in reads	average read size	number of reads	bases in reads	average read size
Arabidopsis	120 Mb	23 M	6919 M	300 bp	481 K	2748 M	5713 bp
Human	3.09 Gb	264 M	39.1 G	300 bp	3.9 M	30.5 G	7821 bp
Chimp	3.31 Gb						

### Genome-to-genome alignment

The primary usage scenario for nucmer3 was to align two genome assemblies or two reference genomes. In this section we demonstrate improvements in timings for such alignments due to parallelization in nucmer4. We use several pairs of plant and animal genomes. Table 3 summarizes the timings and memory usage for the alignments that we ran. In addition to nucmer version 3 & 4, we ran the Mauve [6] and LASTZ [7] whole genome aligners that utilize data structures similar to MUMmer. We aborted all runs after 2 days, and if an aligner took longer, we list the run time as > 2 days. Since it is not straightforward to measure aligner’s accuracy in whole genome alignment, we instead measured sensitivity, which is computed as the proportion of the sequence in the alignments between reference and query, not counting the indels. We found that when using default settings, LASTZ is more sensitive than Nucmer4, but runs much more slowly (“LASTZ default” in the table). Thus, in a second experiment, we adjusted the parameters of LASTZ to approximately match the sensitivity of Nucmer4 with default settings (“LASTZ match”). Nucmer3 was unable to align the chimp reference to the human reference due to limitation in the size of the reference sequence (max 500 Mbp). Nucmer4 peak memory usage is higher both due to its 48-bit index, and due to loading 32 large query sequences at a time for parallel processing, but it runs significantly faster than Nucmer3. Below we provide details on the nucmer4 alignments.

**Table 3:** Timing and memory usage to align two genome sequences for nucmer3 and nucmer4 compared to Mauve and Lastz aligners built using similar data structures. We list both wall clock time and CPU time to show how effective is the code in utilizing multiple cores. Nucmer 4 is the fastest, but not the most memory efficient aligner. Nucmer3 failed to align human to chimp assembly due to the restriction on the size of the reference sequence. LASTZ and Mauve runs on human to chimp alignments took over two days, and we stopped them after that. LASTZ defaults are optimized for high sensitivity, resulting in slow performance. Thus for fairness of timing comparisons we ran LASTZ twice: once with default settings and once with parameters that result in sensitivity matching that of nucmer4 with default settings. We list the parameters in the supplement.

		Arabidopsis	Tardigrade	Human/Chimp
nucmer3	Wall time (min)	17.5	19.6	fail
	CPU time (min)	17.1	19.2	fail
	Memory (GB)	2.1	2.3	fail
nucmer4	Wall time (min)	3.7	4.0	207
	CPU time (min)	22	26	2897
	Memory (GB)	4.6	4.9	66
Mauve	Wall time (min)	41	273	> 2 days
	CPU time (min)	38.6	268	> 2 days
	Memory (GB)	3.3	4.0	> 2 days
LASTZ default	Wall time (min)	1122	> 2 days	> 2 days
	CPU time (min)	1113	> 2 days	> 2 days
	Memory (GB)	1.3		
LASTZ match	Wall time (min)	66	77	> 2 days
	CPU time (min)	66	76	> 2 days
	Memory (GB)	0.6	0.4	

#### Human versus chimp

First we aligned the current assemblies of human and chimpanzee, using the default nucmer4 options with 32 parallel threads. The alignment took 3 hours and 6 minutes on our 32-core Opteron system, and it used a maximum of 66 GB of RAM. Note that Nucmer3 cannot perform an alignment this large unless one first breaks both genomes into smaller pieces. We used human as the reference and chimpanzee as the query sequence. The human GRCh38 assembly contains 3.088 Gb of sequence while chimpanzee assembly, with 3.31 Gb, contains 7% more DNA. (Note that the chimpanzee genome is far less polished than human, and much of the extra DNA might be explained by haplotype variants or incompletely merged regions; thus the two genomes might be much closer in size than these numbers indicate.)

MUMmer had 2.782 Gb of the sequence in mutual best alignments, where each location in the chimp was aligned to its best hit in human and vice versa, with an average identity of 98.07%. The 1.93% nucleotide-level divergence found here is higher than the 1.23% reported in the original chimpanzee genome paper [25]. Our higher divergence is likely due to two factors: first, the 2005 report was based on 2.4 Gb of aligned sequence from older versions of both genomes, while ours is based on 2.782 Gb (16% more sequence) aligned between the current, more-complete versions of both genomes. Second, the original report used different methods, and may have counted fewer small indels than were counted in our alignments. Approximately 306 Mb (9.91%) of the human sequence did not align to the chimpanzee sequence, while 138 Mb (4.15%) of the chimpanzee sequence did not align to human. We detected 390 Mb in alignments where multiple sequences from chimpanzee aligned to the same location in human sequence and thus only one was chosen as the best alignment based on alignment identity. The genomes are very similar across all chromosomes, with the percent identity varying only slightly, from 97.5% to 98.2% for chromosomes 1-22 and X. Chromosome Y was an outlier at 96.6% identity over 84.6% of its length; however this is likely due to the fact that the chimpanzee Y chromosome is much less complete than the human Y.

#### Two *Arabidopsis* species

The alignment of the 207 Mb *A. lyrata* genome to the 120 Mb *A. thaliana* reference genome with nucmer4 took 3m43s on our test system with default parameters using 32 threads. The same alignment performed with nucmer3—which is single threaded— took 17m32s, about 4.7 times slower. These times include the time taken to build the suffix tree or array. We checked the alignments and they were identical between the two programs, as expected. The alignment determined that there are 68.3 Mb in 48,852 1-to-1 best alignments between these two genome sequences with average identity of 88.2%. Overall there are 83 Mb of sequence in 70,044 many-to-many alignments with the same average identity of 88.2%.

#### Two assemblies of a tardigrade genome

A pair of recent studies [26, 27] described the assembly of a microscopic animal, the tardigrade (*Hypsibius dujardini*). The first study described a 212 Mb assembly (here designed Hd-Boothby) and used the results to argue for extensive horizontal gene transfer from unrelated species [26]. The second study, which appeared a few months later, contradicted the first and described a 135 Mb assembly (Hd-Blaxter) that showed little or no evidence for horizontal gene transfer. We aligned the two assemblies using nucmer4, which took 4 minutes, and compared the results using the dnadiff program, which is part of the MUMmer package (see Table 3).

The alignment results showed that 66.5 Mb in Hd-Boothby, the larger assembly, failed to align to Hd-Blaxter. Conversely, 15.9 Mb in Hd-Blaxter failed to align to Hd-Boothby. Given that contamination was an issue of concern for both assemblies, as explained in detail in [27], the nucmer4 output immediately points out which contigs deserve further scrutiny. The results here are consistent with the hypothesis from [27] that Hd-Boothby has far more contamination than Hd-Blaxter. Nucmer also revealed that 109 Mb from Hd-Blaxter aligned to 167 Mb from Hd-Boothby, suggesting that the larger assembly contains many duplicated sequences as compared to the smaller one. While the nucmer alignments do not prove that one assembly is superior to the other, they do allow investigators to quickly identify the regions where assemblies disagree, which enables further analyses to determine which one is correct.

### Alignment of reads to a reference

We can also use nucmer4 to align raw reads to a genome, by providing the genome as a reference and building the suffix array index on that. For optimal speed, one can build the index once and save it as a file (explained above), a new feature in MUMmer4. The suffix array is considerably larger than the compressed Burrows-Wheeler transform used by Bowtie2 and BWA, but the alignment speed is comparable and sometimes faster.

Table 4 compares nucmer4’s performance to several state-of-the-art NGS aligners on the task of aligning both Illumina and PacBio reads to the 120 Mb *Arabidopsis thaliana* genome. Timings were measured on the 32-core AMD Opteron system mentioned above. Default parameters were used for most alignments, except that “-x pacbio” was used for BWA mem with PacBio data (as instructed by the BWA-MEM documentation), and “-l 15 -c 31” was used for nucmer4. We recommend these modifications to the default nucmer4 parameters for long reads with error rates above 10%.

**Table 4:** Timing and memory usage to align PacBio and Illumina reads to the *Arabidopsis thaliana* reference genome. Timings reported here include the time used to build the genome index. The alignments reported by nucmer3 and nucmer4 for the Illumina data were identical. Nucmer3 experienced a reproducible crash when aligning PacBio reads to the *A. thaliana* reference.

	PacBio				Illumina			
	time (min)	memory (MB)	aligned (Mbp)	aligned reads	time (min)	memory (MB)	aligned (Mbp)	aligned reads
blasr	95	4065	1780	435888				
bwa-mem	49	2162	1944	420912	30	3360	6112	21874366
bowtie2					24	686	5580	18716070
nucmer3	fail	fail	fail	fail	334	4688	5651	19873013
nucmer4	24	5743	1713	424271	29	1283	5651	19873013

To align 481,000 PacBio reads to the Arabidopsis genome, nucmer4 took 24 minutes versus 49 minutes for BWA-MEM and 95 minutes for BLASR (Table 4), although nucmer4 required more memory, 5.7 GB versus 4.1 GB (BLASR) and 2.1 GB (BWA-MEM). Nucmer4 aligns about 4% fewer bases than BLASR and about 12% fewer than BWA-MEM, suggesting that BWA-MEM and BLASR are slightly more sensitive, although for this data set we cannot evaluate the correctness of these alignments. To verify the correctness of the alignments, we had to rely on simulated data, in which we know precisely where each read originated. In Table 5 we show results on simulated 1x human genome coverage and 10x Arabidopsis thaliana genome coverage, both generated using the pbsim software [28]. We list the command line that we used to simulate the reads in the Supplementary material. We aligned the faux reads with BLASR, nucmer4 and BWA to the corresponding reference genomes. We found nucmer4 is indeed less sensitive, primarily due to its default behavior of using only unique seeds in the reference. This behavior can be modified with the “--maxmatch” switch at the expense of run time. Nucmer4 also has marginally higher false alignment rate. The sensitivity numbers are consistent with the results on real data.

**Table 5:** Performance of Nucmer4, BLASR and BWA MEM on data simulated by pbsim from human and Arabidopsis reference genomes. All numbers are percentages from the total of bases that are in the reads aligned correctly, missed, or aligned incorrectly. The numbers may not add to exactly 100 due to rounding.

	Arabidopsis			Human		
	Aligned Correctly	Missed	Aligned Incorrectly	Aligned Correctly	Missed	Aligned Incorrectly
nucmer4	94.0	3.5	2.5	84.4	10.9	4.6
blasr	98.2	0.2	1.7	91.8	5.0	3.2
bwa-mem	98.7	0.5	0.8	91.6	5.9	2.5

For aligning Illumina reads to Arabidopsis (Table 4), nucmer4 was about 30 times faster than Nucmer3 and slightly faster than BWA, but about 20% slower than Bowtie2. Nucmer4 aligned more reads and more sequence than Bowtie2 but about 10% fewer than BWA. For each program, the time reported in Table 4 is the sum of the time to create the index and to align the reads. Because this is a small reference genome (∼120 Mbp) with about 100x Illumina coverage, the amount of time to create the suffix array or the Burrows-Wheeler index was negligible compared to the time to create the alignments. Nucmer4 used about twice as much memory (1.3 GB) as Bowtie2 (0.69 GB), but less than half as much as BWA (3.4 GB).

The creation of a Burrows-Wheeler index or a suffix array is costly for a large genome such as the 3 Gb human genome. Thus it is common practice to precompute the index and simply load it into memory at the time of alignment. For alignments of reads to the human genome, therefore, we report separately the time to create the index and to align the reads.

Table 6 shows, not surprisingly, that nucmer4 uses much more memory (45.1 GB) than either BWA-MEM (11.2 GB) or Bowtie2 (4.0 GB). However, the 45 GB required for nucmer4 is readily available on contemporary server-class computers. For these data sets (264 M Illumina reads and 3.9 M PacBio reads), Nucmer4 was the fastest program for aligning both Illumina and Pacbio reads, about 10% faster than Bowtie2 and 30% faster than BWA-MEM. Thus by using more memory, nucmer4 makes a trade-off that results in substantially increased speed. Nucmer4 is less sensitive, it aligns 3-5% fewer reads than BWA or Bowtie2, likely due to two reasons. First, it uses relatively long exact matches to seed its alignments. With its default setting used here, nucmer4 used 20-base (or longer) exact matches to seed the alignments, while Bowtie2 and BWA-MEM use shorter seed lengths. Second, by default, the seeds must be unique in the reference sequence, thus no seeds will be found in sub-sections of the reference sequence longer than the seed length, that are present in multiple copies. This behavior can be changed with the “--maxmatch” switch, which will force nucmer4 to use all seeds at the cost of longer run times.

**Table 6:** Timing and memory usage to align Illumina and PacBio reads to human reference.

	Illumina reads to Human reference					
	build index		align		result	
	time (min)	memory (GB)	time (min)	memory (GB)	aligned bases (Gbp)	aligned reads
bwa-mem	96	4.5	197	11.2	38.46	263155221
bowtie2	51	18.6	163	4.0	38.00	258560571
nucmer4	36	45.1	146	45.5	36.71	250689492
	PacBio reads to Human reference					
	build index		align		result	
	time (min)	memory (GB)	time (min)	memory (GB)	aligned bases (Gbp)	aligned reads
blasr	40	29.4	1680	47.9	24.41	3836927
bwa-mem	96	4.5	1473	7.7	25.86	3820163
nucmer4	36	45.1	850	50.1	23.02	3784039

#### Parallelization

**Fig 1:**
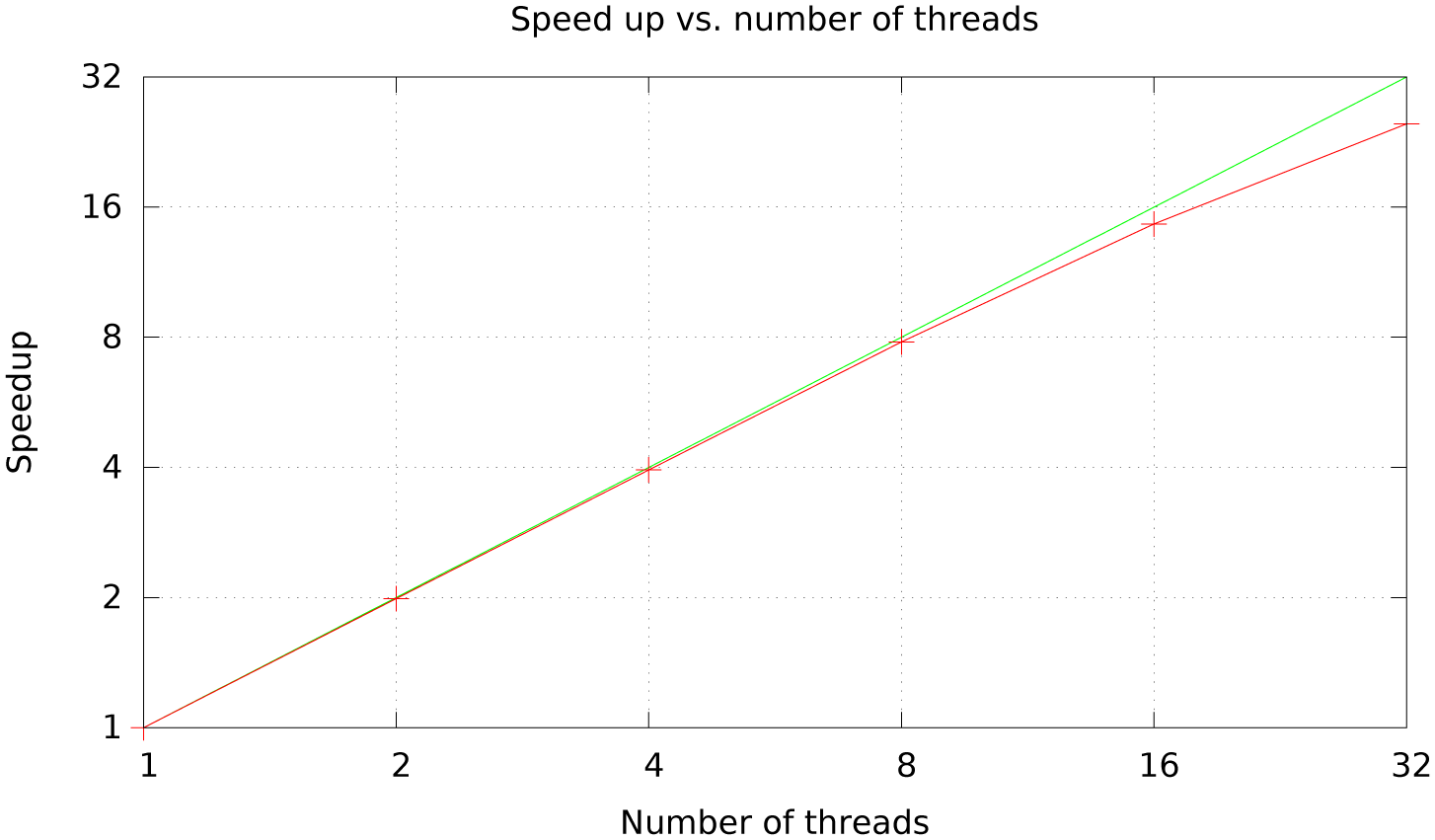
Scaling of nucmer4’s performance when aligning Illumina reads to the *A. thaliana* genome with 1–32 threads. All tests were run on a 32-core AMD Opteron computer.

Fig 1 shows that Nucmer4’s alignment speed scales almost linearly from 2 to 16 parallel computing threads, and becomes sublinear at about 32 threads or more. The deviation from linear performance is likely due to the inherent random memory access for the suffix array where multiple threads start competing, saturating the memory bandwidth in our AMD Opteron system.

## Availability

The MUMmer4 software including source code is available under an open source license at http://mummer4.github.io.

## Future directions

In this paper we described MUMmer4, the successor to MUMmer3, a versatile and efficient genome alignment system. Nucmer4, the primary DNA sequence aligner in the MUMmer4 package, can be used for a variety of tasks ranging from simple alignment of two genome sequences to alignment of large, complex draft genomes with thousands of contigs. With the performance enhancements in this new system, Nucmer4 can align two mammalian genomes in about three hours on a 32-core server; we illustrated this by using it to compute that almost 90% of the human genome is about 98% identical to the chimpanzee genome.

Our future plans include support and maintenance of the software and improvements to other useful parts of MUMmer such as Promer, the protein sequence aligner.

## Supporting information

S1 File Supplemental information. (DOCX)
