# Supplementary material for: MUMmer4: A fast and versatile genome alignment system
Source: PLoS Comput Biol. 2018 Jan 26;14(1):e1005944. doi: 10.1371/journal.pcbi.1005944 (PMC5802927; doi:10.1371/journal.pcbi.1005944)
Supplement: S1 File — (DOCX) [file pcbi.1005944.s001.docx]

**Supplemental information.**

**Analysis of alignments of simulated data.**

We used human and Arabidopsis reference genomes to generate 1x and 10x respective coverage of the genomes with simulated reads. We used the following commands to generate simulated reads

./PBSIM-PacBio-Simulator/src/bin/Debug/PBSIM --depth 10 --model_qc ./PBSIM-PacBio-Simulator/data/model_qc_clr Arabidopsis.fa

./PBSIM-PacBio-Simulator/src/bin/Debug/PBSIM --depth 1 --model_qc ./PBSIM-PacBio-Simulator/data/model_qc_clr HS.fa

**Accession numbers for the assemblies used in the paper**

1. Human: GCA_000001405.22
2. Chimpanzee: GCF_000001515.6
3. Tardigrade:
   1. Hd-Boothby GCA_001455005.1
   2. http://ensembl.tardigrades.org/Hypsibius_dujardini_nhd315/Info/Index
4. Arabidopsis
   1. A.thaliana: GCA_000001735.1
   2. A.lyrata: GCA_000004255.1

**Measurement of the timings.**

We ran all our timings on a 32-core AMD Opteron 6276 2.3GHz computer with 256Gb of DDR3-1066 RAM.

**Recommended parameters for running various tasks with nucmer:**

- 1. Genome-to-genome alignment: default parameters
  2. Illumina reads to genome alignment: default parameters
  3. Pacbio reads to genome alignment: -l 15 -c 31

**Command line parameters used for the timing runs:**

Genome-to-genome alignments:

Nucmer3:
Nucmer4: -t 32
LASTZ: --exact=20 --maxwordcount=1 --notransition --step=5 --format=maf <target.fa>[multiple,format=fasta] <query>
Mauve:

Pacbio-to-genome alignments:

Blasr: -minMatch 15 -nproc 32
 Bwa: mem -x pacbio -t 32
 Nucmer4: -l 15 -c 31

Illumina-to-genome alignments:

Bowtie2: --threads 32
 Bwa: mem -t 32
 Nucmer 4: -t 32

**Use the library and bindings of Nucmer: align two sequence strings**

The following examples of code show how to align two sequences against each other in C++ and in the script languages Ruby, Perl and Python. All these examples are also available in the source code repository, with instructions on how to compile and use them. All examples create an ‘Options’ object which contains the many parameters that control the alignment process. Then, they call the function ‘align_sequences’ which takes two strings containing the sequences and the option object previously create.

A list (as a vector or array) of alignments is returned. Each alignment contains the position of the alignment in the reference and query coordinates, information on the quality of the alignment and the delta array, giving the relative position of the indels (as described in the delta format).

C++:

| #include <iostream>  #include <fstream>  #include <mummer/nucmer.hpp>  std::string read_file(const char* path) {  std::ifstream is(path);  std::string res, line;  while(std::getline(is, line))  res += line;  return res;  }  int main(int argc, char *argv[]) {  std::string ref = read_file(argv[1]);  std::string qry = read_file(argv[2]);  mummer::nucmer::Options o;  o.minmatch(10).mincluster(10);  auto aligns = mummer::nucmer::align_sequences(ref.c_str(), ref.size(), qry.c_str(), qry.size(), o);  for(const auto& a : aligns) {  std::cout << a.sA << ' ' << a.eA << ' ' << a.sB << ' ' << a.eB << ' '  << a.Errors << ' ' << a.SimErrors << ' ' << a.NonAlphas << '\n';  for(auto d : a.delta)  std::cout << d << '\n'; std::cout << "0\n";  }  return 0;  } |
| --- |

Ruby:

| # /usr/bin/env ruby  require "mummer"  ref = File.read(ARGV[0])  qry = File.read(ARGV[1])  o = Mummer::Options.new.minmatch(10).mincluster(10)  aligns = Mummer::align_sequences(ref, qry, o)  aligns.each { \|a\|  puts("#{a.sA} #{a.eA} #{a.sB} #{a.eB} #{a.Errors} #{a.SimErrors} #{a.NonAlphas}")  puts(a.delta.join("\n")) unless a.delta.empty?  puts("0")  } |
| --- |

Perl:

| #! /usr/bin/env perl  sub read_file {  local $/ = undef;  my ($file) = @_;  open(FILE, "<", $file) or die "Can't open file '$file': $!";  binmode FILE;  my $s = <FILE>;  close(FILE);  return $s;  }  my $ref = read_file($ARGV[0]);  my $qry = read_file($ARGV[1]);  my $o = mummer::Options->new;  $o->minmatch(10);  $o->mincluster(10);  my $aligns = mummer::align_sequences($ref, $qry, $o);  print($aligns, "\n");  for my $a (@$aligns) {  print("$$a{sA} $$a{eA} $$a{sB} $$a{eB} $$a{Errors} $$a{SimErrors} $$a{NonAlphas}\n");  print("0\n");  } |
| --- |

Python:

| #! /usr/bin/env python  import mummer  import sys  fd = open(sys.argv[1])  ref = fd.read()  fd.close()  fd = open(sys.argv[2])  qry = fd.read()  fd.close()  o = mummer.Options()  o.minmatch(10)  o.mincluster(10)  aligns = mummer.align_sequences(ref, qry, o)  for a in aligns:  print("%d %d %d %d %d %d %d" % (a.sA, a.eA, a.sB, a.eB, a.Errors, a.SimErrors, a.NonAlphas))  for d in a.delta:  print(d)  print("0") |
| --- |
